# lncRNAKB, a knowledgebase of tissue-specific functional annotation and trait association of long noncoding RNA

**DOI:** 10.1038/s41597-020-00659-z

**Published:** 2020-10-05

**Authors:** Fayaz Seifuddin, Komudi Singh, Abhilash Suresh, Jennifer T. Judy, Yun-Ching Chen, Vijender Chaitankar, Ilker Tunc, Xiangbo Ruan, Ping Li, Yi Chen, Haiming Cao, Richard S. Lee, Fernando S. Goes, Peter P. Zandi, M. Saleet Jafri, Mehdi Pirooznia

**Affiliations:** 1 Bioinformatics and Computational Biology Core, National Heart, Lung, and Blood Institute, National Institutes of Health, Bethesda, MD 20892 USA; 2 Cardiovascular Branch, National Heart, Lung and Blood Institute, National Institutes of Health, Bethesda, MD 20892 USA; 3 Department of Psychiatry & Behavioral Science, The Johns Hopkins University School of Medicine, Baltimore, MD 21205 USA; 4 School of Systems Biology, George Mason University, Manassas, VA 20110 USA; 5 Krasnow Institute for Advanced Study, Interdisciplinary Program in Neuroscience, George Mason University, Fairfax, VA 22030 USA

**Keywords:** Data processing, Functional clustering, Genetic databases, Data integration

## Abstract

Long non-coding RNA Knowledgebase (lncRNAKB) is an integrated resource for exploring lncRNA biology in the context of tissue-specificity and disease association. A systematic integration of annotations from six independent databases resulted in 77,199 human lncRNA (224,286 transcripts). The user-friendly knowledgebase covers a comprehensive breadth and depth of lncRNA annotation. lncRNAKB is a compendium of expression patterns, derived from analysis of RNA-seq data in thousands of samples across 31 solid human normal tissues (GTEx). Thousands of co-expression modules identified via network analysis and pathway enrichment to delineate lncRNA function are also accessible. Millions of expression quantitative trait loci (*cis*-eQTL) computed using whole genome sequence genotype data (GTEx) can be downloaded at lncRNAKB that also includes tissue-specificity, phylogenetic conservation and coding potential scores. Tissue-specific lncRNA-trait associations encompassing 323 GWAS (UK Biobank) are also provided. LncRNAKB is accessible at http://www.lncrnakb.org/, and the data are freely available through Open Science Framework (10.17605/OSF.IO/RU4D2).

## Abstract (summary)

**Table:** 

Measurement(s)	regulation of gene expression • sequence feature annotation • lnc_RNA • tissue-specific expression of lncRNA • Expression Quantitative Trait Locus
Technology Type(s)	digital curation • computational modeling technique
Sample Characteristic - Organism	Homo sapiens

Machine-accessible metadata file describing the reported data: 10.6084/m9.figshare.12827597

## Background & Summary

While 70–90% of the mammalian genome is transcribed into RNA, only 1% of the genome is directly translated into protein, leaving the majority of transcripts as non-coding RNA (ncRNA). Once dismissed as ‘transcriptional noise’, results from high-throughput RNA analyses have shifted the paradigm towards an increasing appreciation for likely regulatory role^1^, including potential roles in many biological processes including transcriptional and post-transcriptional regulation, epigenetic regulation, organ or tissue development, cell differentiation and apoptosis, cell cycle control, cellular transport, metabolic processes and chromosome dynamics^2,3^. Long non-coding RNA (lncRNA) are a specific type of these regulatory transcripts defined by size that ranges from 200 base pairs (bp) to 100 kilobases (kb)^4^ in length. Notable features of lncRNA include minimal interspecies conservation^5–8^, with conserved sequences generally confined to short, 5′-biased patches of conserved sequences nested in exons^5^, and a relatively higher degree of tissue-specific expression as compared to mRNA^6,9^. Some lncRNA undergo translation with a low level of expression^2^, though only a minority of such translation events results in stable and functional peptides^10–12^.

Several publicly available resources dedicated to annotation of lncRNA in humans and other species have been developed as shown in Table 1^13–31^. Most of these databases are available through web-based searchable interfaces and provide downloadable annotation files in Gene Feature Format (GFF)^27,32,33^ or Gene Transfer Format (GTF) thereby, allowing users to quantify the lncRNA expression patterns of their own sequence data. Some of these databases incorporate additional genomics data on lncRNA, including expression, methylation, variation, conservation and functional annotation. Commonly cited resources of lncRNAs annotation (GFF) include GENCODE^29,34^, CHESS^18^, LNCipedia^19,20^, NONCODE^21^, FANTOM^35^, MiTranscriptome^25^ and BIGTranscriptome^26^. These resources annotate lncRNA by two approaches: manual or automatic^13^. Manual annotation involves human annotators curating gene and transcript models based on RNA and protein experimental evidence and defined sets of rules^29^. Automatic annotation uses bioinformatics methods such as StringTie^36^ and Cufflinks^37^ to reconstruct gene and transcript models based on billions of short RNA-sequence (RNA-seq) reads^25^. Although many lncRNA databases exist, a consolidated resource that leverages the synergy of their individual strengths is lacking, hindering efforts to systematically identify lncRNA relevant to human traits using current analysis methods and large genomics data.

**Table 1:** Resources of human lncRNA annotation.

Database Name	Reference build	Annotation file name	URL
CHESS^18^	hg38	chess2.2.gtf	http://ccb.jhu.edu/chess/data/chess2.2.gtf.gz
LNCipedia^19,20^	hg19,hg38	lncipedia_5_2_hc_hg38.gtf	https://lncipedia.org/downloads/lncipedia_5_2/full-database/lncipedia_5_2_hg38.gtf
NONCODE^21^	hg19,hg38	NONCODEv5_human_hg38_lncRNA.gtf	http://www.noncode.org/datadownload/NONCODEv5_human_hg38_lncRNA.gtf.gz
FANTOM5^22^	hg19	FANTOM_CAT.lv3_robust.only_lncRNA.gtf	https://fantom.gsc.riken.jp/5/suppl/Hon_et_al_2016/data/assembly/lv3_robust/FANTOM_CAT.lv3_robust.only_lncRNA.gtf.gz
MiTranscriptome^25^	hg19	mitranscriptome.hg19.v2.gtf	http://mitranscriptome.org/download/mitranscriptome.gtf.tar.gz
BIGTranscriptome^26^	hg19	BIGTranscriptome_lncRNA_catalog.hg19.gtf	http://big.hanyang.ac.kr/UCSC/RNA-seq/hg19/CAFE/GTFs/BIGTranscriptome/BIGTranscriptome_lncRNA_catalog.gtf
deepBase^23^	hg19	hg19_allLncRNA.rnaFam.bed	http://rna.sysu.edu.cn/deepBase/Download/hg19_allLncRNA.rnaFam.bed
lncRNAdb^17^	hg38	under development	http://lncrnadb.org/
LncRNAWiki^24^	hg19	RawData.tar.gz	http://lncrna.big.ac.cn/data/RawData.tar.gz
LncBook^27^	hg19,hg38	LncBook_GENCODE_GRCh38_9.28.gtf.gz	ftp://download.big.ac.cn/lncbook/1-LncRNAs(GRCh37%7C38)/LncBook_GENCODE_GRCh38_9.28.gtf.gz
RNAcentral^28^	hg38	homo_sapiens.GRCh38.gff3.gz	ftp://ftp.ebi.ac.uk/pub/databases/RNAcentral/releases/14.0/genome_coordinates/gff3/homo_sapiens.GRCh38.gff3.gz
GENCODE^29^	hg19,hg38	gencode.v33.annotation.gtf.gz	ftp://ftp.ebi.ac.uk/pub/databases/gencode/Gencode_human/release_33/gencode.v33.annotation.gtf.gz
ENSEMBL^30^	hg19,hg38	Homo_sapiens.GRCh38.99.gtf.gz	ftp://ftp.ensembl.org/pub/release-99/gtf/homo_sapiens/Homo_sapiens.GRCh38.99.gtf.gz
RefSeq.^31^	hg19,hg38	GRCh38_latest_genomic.gtf.gz	ftp://ftp.ncbi.nlm.nih.gov/refseq/H_sapiens/annotation/GRCh38_latest/refseq_identifiers/GRCh38_latest_genomic.gtf.gz

We developed lncRNAKB by rigorously combining annotations from the frequently used lncRNA databases mentioned above using a cumulative stepwise intersection method. Our method of integration systematically compiled lncRNA annotations from each source, eliminating ambiguous and redundant records. The resulting knowledgebase is a comprehensive, downloadable, searchable and viewable (via the UCSC Genome Browser)^38^ GFF annotation file of human protein-coding genes (PCGs) and a large number of lncRNA (*n* = 77,199).

We then proceeded to apply this master annotation to the following subsequent features of the knowledgebase. We implemented an up-to-date analysis pipeline processing RNA-Seq data available through the Genotype Tissue Expression (GTEx Release v7) project^39^, and then quantified expression via a body map of human lncRNA across 31 solid normal human tissues (gene and transcript level). Using gene expression information, we calculated tissue-specificity scores. To explore the impact of genotype variants on expression, we then calculated expression quantitative trait loci (eQTL) using the GTEx expression and whole genome sequencing (WGS) genotype data, providing a tissue-specific eQTL body map of lncRNA. LncRNAKB includes information on classification of lncRNA based on their positional information and coding potential using FlExible Extraction of LncRNAs (FEELnc)^40^ algorithm. Furthermore, it provides exon-level conservation scores derived from an alignment of 30 vertebrate species^38^. We used Weighted Gene Co-expression Network Analysis (WGCNA)^41^ method to analyze lncRNA-mRNA co-expression patterns in a tissue-specific manner to support prediction of lncRNA functions. The co-expression modules were further investigated via pathway enrichment analysis to identify functional pathways associated with lncRNA. Moreover, for each tissue we manually selected 25 notable pathways (with some biological relevance to the tissue of interest) and created a dynamic network figure on the website to view the strength of connections between strongly correlated mRNA and lncRNA. Finally, lncRNA-trait associations were tested using 323 traits from the UK Biobank^42^ (>5,000 cases) across all tissues via summary mendelian randomization (SMR)^43^ analysis. Data from all analysis are available in the knowledgebase at http://www.lncrnakb.org/. In addition, the data are freely available through Open Science Framework (https://doi.org/10.17605/OSF.IO/RU4D2)^44^.

## Methods

**Fig. 1:**
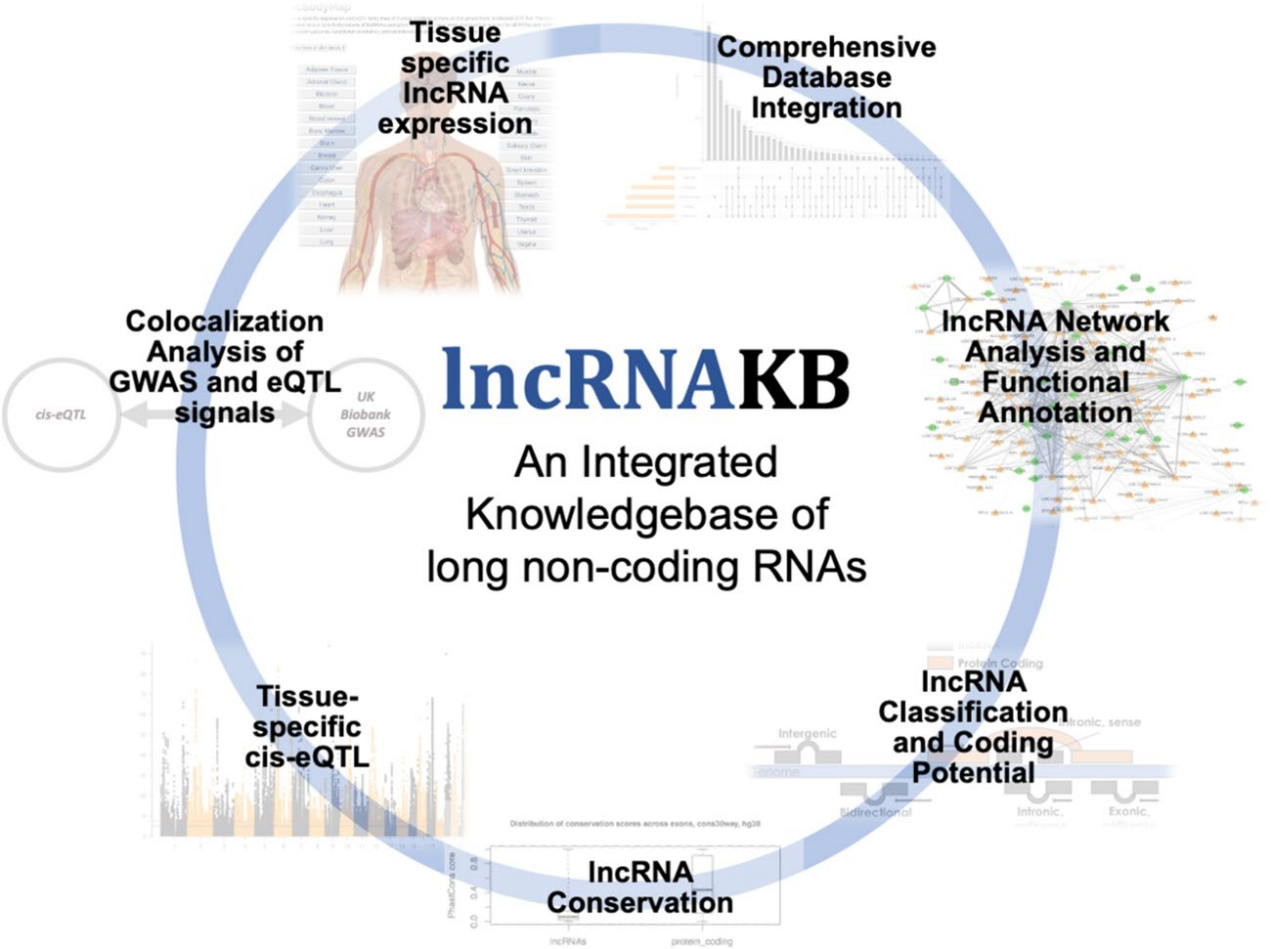
Overview of lncRNAKB.

lncRNAKB is an integration of six lncRNA annotation databases. The resulting knowledgebase considers lncRNA data from many perspectives, including quantitation of expression with GTEx RNA-Seq data, tissue specificity, consideration of eQTL, co-expression with protein coding genes and subsequent network analysis for functional characterization, and finally, lncRNA-trait associations with hundreds of disease phenotypes from the UK Biobank GWAS data. Figure 1 illustrates the overview of lncRNAKB.

### Integration of lncRNA annotation databases

To identify widely used lncRNA annotations and databases for integration into the knowledgebase, we performed a literature search of the PubMed database through February 28^th^, 2019 with the following keyword algorithm: *(lncrna or long noncoding or long non-coding rna or noncoding) and (annotation or function or database)*. Results were filtered by human species and limited to publications within the past five years, in English, then sorted by the best match criteria. A total of 13,412 articles were returned. The titles, abstracts, keywords, and full text were manually reviewed (divided amongst four reviewers) to identify publications that reported lncRNA annotations, databases and function. The references of these articles were also searched to identify other articles that were potentially missed by the initial PubMed search. For inclusion in the review, the study had to be an RNA-Seq study, used a GFF annotation to quantify the data and mentioned lncRNA in their results. After this review, six lncRNA databases were selected for step-wise integration to create a single lncRNA annotation for lncRNAKB. The six resources are: CHESS (version 2.1), LNCipedia (v5.2), NONCODE (v5.0), FANTOM (5.0.v3), MiTranscriptome (v2) and BIGTranscriptome (v1).

The GFF annotation files from all six databases (links in Table 1) were downloaded. To streamline the data integration step, all the GFF annotations were parsed to the same format using the following steps:

(i) All GFF files were required to be annotated according to hg38 (the latest genome build). Annotations to the previous build (hg19) were updated using the UCSC liftOver tool^38^ from hg19 to hg38.
(ii) The gene and transcript records were split into individual files by chromosome, and labelled with location, including chromosome, strand, start and end base pair locations. Each gene block file contained the transcripts information and the transcript block file contained the exons information. In cases where the transcripts or exons records lacked genes information, a gene entry was manually created using the gene ids in the transcripts or exons records and combined with the base pair locations of the first exon (as gene start), of the last exon (as gene end), and transcript strand to represent the gene strand. All redundant records (genes and corresponding transcripts with the same exonic start and end coordinates) between annotation files were removed in this process.

**Fig. 2:**
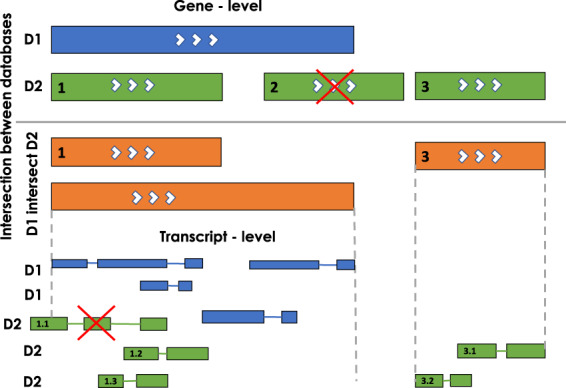
Illustration showing the stepwise intersection of two annotations D1 (CHESS) (blue) and D2 (FANTOM-lncRNAs only) (green) at the gene and transcript levels. The genes are shown as solid rectangles and the transcripts are shown with exons and introns. The white arrows show the direction/strand in which the gene is transcribed. The orange bars show the results of the intersection (D1 intersect D2) at the gene level. The red X marks show transcripts and genes that were not incorporated into the merged annotation. D3 (LNCipedia), D4 (NONCODE), D5 (MiTranscriptome) and D6 (BIGTranscriptome) were merged using the same cumulative stepwise intersection method (see Methods: Integration of lncRNA annotations).

Using CHESS (contains virtually all genes from RefSeq (as of mid-2017) and GENCODE) as the reference annotation (containing both protein-coding and lncRNA genes) we used a cumulative stepwise intersection method to merge it with the rest of the five lncRNA annotations in the following order: (i) FANTOM, (ii) LNCipedia, (iii) NONCODE, (iv) MiTranscriptome and (v) BIGTranscriptome at the genes and transcripts levels. This order of intersection was chosen based on experimental evidence for lncRNA in individual annotations. Figure 2 illustrates the cumulative stepwise intersection method for two annotations as an example, D1 (CHESS) in blue and D2 (FANTOM-lncRNA only) in green. For each gene entry in D1 (top blue panel), we kept genes from D2 (green panel) that had full overlap or enclosed within D1’s gene boundary (labelled as 1) or outside the boundaries of D1 (labelled as 3). The resulting intersection is shown in orange. D2’s gene that had partial overlap with D1’s gene (labelled as 2 and marked with a red X) was discarded as we did not want to re-define gene boundaries in the reference annotation.

For genes that intersected, the transcript records (shown as smaller bars connected by lines to represent exons and introns, respectively) from D1 and D2 were compared. Similarly, to the gene intersection, transcript entries whose start and end were within the gene boundaries were included (labelled as 1.2, 1.3, 3.1 and 3.2). Several transcripts (labelled as 1.1 and marked with a red X) that fell outside the gene boundary and were probably incorrectly assigned to genes were removed in this process. In addition, if a transcript in D2 had partial or no overlap with transcripts in D1, we incorporated that transcript (labelled as 1.2 and 1.3) including all the exons to the gene record accordingly. For genes with no overlap in D1, we added all the transcripts and corresponding exons to the merged annotation as a lncRNA entry (labelled as 3.1 and 3.2).

### Expression profiling

To quantify gene expression patterns of the consolidated lncRNA records, we queried RNA-seq data available through the Genotype Tissue Expression (GTEx Release v7) project^39^. We downloaded the raw paired-end RNA-seq data (FASTQ files) from the dbGap portal (study_id = phs000424.v7.p2) of 31 solid human normal tissues. For each tissue, quality control of paired-end reads were assessed using FastQC tools^45^, adapter sequences and low-quality bases were trimmed using Trimmomatic^46^ and aligned to the human reference genome (*H. sapiens*, GRCh38) using HISAT2^47^. Utilizing uniquely aligned reads to the human genome, gene-level expression quantitation (via raw read counts) was generated with the featureCounts software^48^ guided by the lncRNAKB GFF annotation. Transcript-level expression of the lncRNAKB transcripts FASTA file was quantified using Salmon^49^. Based on the distribution of uniquely mapped paired-end reads assigned to genes across all the GTEx samples, samples with <10^6^ reads assigned to genes were excluded. We normalized the raw read counts to Transcripts Per Kilobase Million (TPM)^50^. To explore gene expression similarity between tissues and across GTEx samples as well as summarize lncRNA tissue-specific expression we performed a principal component analysis (PCA) using the prcomp package in R^51,52^. We used the normalized TPM expression values, transformed by taking the *log*_2_(*TPM*), across all lncRNA (*n* = 77,199) and tissues (*n* = 31) (no filters applied).

### Tissue-specificity scores

In addition to gene expression quantitation, we calculated two tissue-specificity metrics (Tau and Preferential Expression Measure (PEM))^53,54^ using the normalized TPM expression values by gene across tissues. Tau summarizes in a single number whether a gene is tissue-specific or ubiquitously expressed across all tissues. PEM shows for each tissue separately how specific the gene is to that tissue. The PEM scores the expression of a gene in a given tissue in relation to its average expression across all other genes and tissues. The average gene expression across all replicates by tissue was used to compute Tau and PEM. Genes that were not expressed in at least one tissue were excluded from the analysis.

### Genotype file processing

The whole genome sequence (WGS) data in blood-derived DNA samples from the GTEx portal (dbGaP: phs000424.v7.p2) was downloaded to conduct tissue-specific expression quantitative trait loci (eQTL) analysis. First, the VCF files were processed using the following steps with a combination of PLINKv1.9^55,56^ vcftools v0.1.15^57^ and bcftools v1.9^58^: (i) remove indels; (ii) exclude missing and multi-allelic variants; (iii) selected “FILTER =  = ‘PASS’“ variants; (iv) exclude variants with minor allele frequency (MAF) <5%; (v) update the coordinates of single nucleotide polymorphisms (SNPs) using the UCSC liftOver tool^38^ from hg19 to hg38 (latest genome build); (vi) change the SNPs IDs to dbSNP^59^ rsID using dbSNP Build 151; (vii) convert to bed, bim and fam format. For each solid tissue, subjects with both WGS data and gene expression data were selected. The VCF file was subset by tissue and the MAF recalculated to exclude variants with MAF <5%. After converting to ped and map format, we ran principal component analysis (PCA) on each tissue to get a set of genotype covariates using eigensoft v6.1.4^51,60^.

### eQTL analysis

For each solid tissue, we implemented a two-step filtering approach, which is similar to the steps adapted by GTEx^39^. Briefly, the genes were first filtered based on TPM to include genes with TPM >0.50 in at least 20% of the samples in each tissue to eliminate the low-expressed genes which obscure meaningful signals with noise. Next, the genes were filtered based on raw counts to include protein-coding genes and non-coding genes with counts >2 and >1 in at least 20% of the samples in each tissue, respectively. The edgeR^61^ and limma-voom^62,63^ package in R^64^ were used to process the filtered read counts into log_2_ counts per million (log_2_CPM) that were normalized using trimmed mean of M-values (TMM)^65^. The expression files were then sorted by gene start and stop, compressed with BGZIP and indexed with TABIX^66^. Only tissues with >80 samples were included in the *cis-*eQTL analysis. For eQTL analysis, the first five principal components (PCs) (see Genotype file processing), sex, genotype platform and 15 probabilistic estimation of expression residuals (PEER) factors^67^ were included as covariates. Within each tissue, *cis*-eQTLs were identified by linear regression, as implemented in FastQTLv2.0 (threaded option)^68^, adjusting for all the covariates. We restricted our search to variants within 1 megabase (Mb) of the boundary (start and end) of each gene. We used the Benjamini and Hochberg correction method^69^ to calculate the false discovery rate (FDR) in R statistical programming language (R)^64^ across all SNP-gene pairs. For each tissue, all *cis*-eQTL results were visualized using a Manhattan plot created using the qqman package in R^70^.

### Functional characterization of lncRNA using a network-based approach

Using the filtered log_2_CPM and TMM normalized gene expression data (see Methods: Expression Profiling), we used the weighted gene co-expression network analysis (WGCNA) approach^41^ as implemented in the Co-Expression Modules identification Tool (CEMiTool) package in R^71^ to identify modules of lncRNA-mRNA clusters that are co-expressed and therefore likely work in concert to carry out various biological functions. For this, the gene expression data was filtered by log_2_CPM >2 in at least 50% of the samples to avoid random correlations between low-expressing genes. The default CEMiTool parameters were used with the following exceptions: (i) Pearson method was used for calculating the correlation coefficients, (ii) the network type used was unsigned, (iii) no additional filter parameters in CEMITool were used for the expression data, (iv) applied Variance Stabilizing Transformation (VST) and the correlation threshold for merging similar modules were set to 0.90. All the co-expressed modules were subjected to over-representation analysis (ORA) by module based on the hypergeometric test^72^. We used Gene Ontology (GO) terms^73–75^ to check for overrepresentation of genes and determined the most significant module functions based on pathways FDR q-value ≤0.05^76^. The background set used for the pathway enrichment analysis was genes represented across all GO terms. To visualize the interactions between the genes in each co-expression module, we manually selected 25 notable pathways (with some biological relevance to the tissue of interest) for each tissue. The module adjacency matrices for each module was filtered based on correlations >0.20 across all genes. A JSON file (one per pathway) was created to produce interactive networks using Cytoscape v3.6.0 JavaScript modules^77^. The network files and the module adjacency/correlation matrix files are available for downloading on lncRNAKB.

### Colocalization analysis of GWAS and eQTL signals

Summary Mendelian Randomization analysis (SMR)^43^ is a method that prioritizes genes that are targeted by genetic variants/SNPs in GWAS of complex diseases. It combines summary-level data from two-samples for e.g. independent GWAS and data from eQTL studies to identify pleiotropic association between the expression level of a gene (exposure) and a trait (outcome). Pleiotropic association is when the causal variant affects both gene expression and trait. SMR and HEIDI (Heterogeneity in dependent instruments) methods implemented in the SMR package^43^ were used to test the association between lncRNA gene expression and traits tested by means of colocalization of summary GWAS and *cis*-eQTL signals. Particularly, HEIDI uses multiple SNPs (*n = *20) in a *cis*-eQTL region to distinguish pleiotropy from linkage, and a pHEIDI >0.05 suggests non-heterogeneity, thus colocalized. Briefly, summary GWAS data for 323 traits with >5,000 cases available in the UK Biobank were downloaded (Figshare File F2)^78^ and formatted into.ma format as specified on the CNS genomics’ website (http://cnsgenomics.com/software/smr/). Results from the eQTL analysis were filtered by FDR ≤0.05 and formatted into BESD format. SMR was then conducted separately using GWAS meta-analyses summary data for each of the 323 traits (Figshare File F2)^78^ using a default cis window of 2000 Kb and p-value of eQTL set to 5 × 10^−4^ for selecting top *cis*-eQTL SNPs in all tissues with eQTL information.

### Evaluation of coding potential of lncRNAs

FlExible Extraction of LncRNAs (FEELnc)^40^ was used to classify/annotate and calculate the coding potential of all the gene entries in the lncRNAKB. FEELnc annotates lncRNAs based on a machine learning method, Random Forest (RF)^79^, trained with general features such as multi *k-*mer frequencies, RNA sequence length and open reading frames (ORFs) size. It is comprised of three modules: (i) filter, (ii) coding potential, and (iii) classifier. The filter module flags and removes transcripts overlapping (in sense) exons of the reference annotation, specifically the protein-coding exons. We used the GENCODEv29^29^ GFF file as the reference annotation to get an estimate of the number of transcripts from lncRNAKB overlapping with “*protein_coding*” transcripts. We set the minimal fraction out of the candidate lncRNAs size to be considered for overlap to be excluded as 0.75 (>75% overlap) to retain many lncRNAs transcripts. Transcripts <200 base pairs (bp) long were filtered out but, monoexonic transcripts were included in the analysis. We then used the filtered GFF annotation output file from the filter module and calculated a coding potential score (CPS) for each transcript using the coding potential module. Due to the lack of a gold standard/known human lncRNAs data set for training, we used the “intergenic” mode in the module. This approach extracts random intergenic sequences of length *L* from the genome of interest to model species-specific noncoding sequences as the non-coding training set. We used the human reference genome FASTA file (hg38) and the GENCODE GFF file as the reference annotation. To get the best training set of known mRNA, we used “*transcript_biotype* = *protein_coding*” and “*transcript_status* = *KNOWN*” for the RF model. We used the default values for the *k*-mer sizes, number of trees and ORF type. To determine an optimal CPS cut-off, FEELnc automatically extracts the CPS that maximizes both sensitivity and specificity based on a 10-fold cross-validation. The CPS was between 0 and 1 where 0 indicates a non-coding RNA and a score close to 1 a mRNA. And finally, to classify potential lncRNA with respect to the localization and the direction of transcription of nearby mRNA (or other non-coding RNAs) transcripts as shown in Figshare File F1^78^, we used the classifier module. We used the final set of lncRNAs transcripts output from the coding potential module and classified them using the GENCODEv29 GFF file as the reference annotation. A sliding window size around each lncRNA was used to check for possible overlap with nearest reference transcripts. We used a minimum and maximum window size of 10 kilobase (kb) and 100 kb respectively. The classification method reported all interactions within the defined window and established a best partner transcript using certain rules.

### Conservation analysis

Conservation of exons between protein-coding genes and lncRNAs in the lncRNAKB annotation database was analyzed using the bigWigAverageOverBed^80^ and the cons30way (hg38) track^81^ both downloaded from the UCSC genome browser. This track shows multiple alignments of 30 vertebrate species and measurements of evolutionary conservation using two methods (phastCons and phyloP^82^) from the PHAST package^83^ for all thirty species. The multiple alignments were generated using multiz^84^ and other tools in the UCSC/Penn State Bioinformatics comparative genomics alignment pipeline. An exon-level BED file was created using the lncRNAKB GFF annotation file separately for protein-coding genes and lncRNAs. We merged overlapping exons within transcripts to avoid counting conservation scores of overlapping base pairs more than once. For each exon, the bigWigAverageOverBed function calculates the average conservation score across all base pairs. Using line graphs, we visualized and compared the average conservation score differences between lncRNAs and protein-coding exons.

### Architecture of the database

**Fig. 3:**
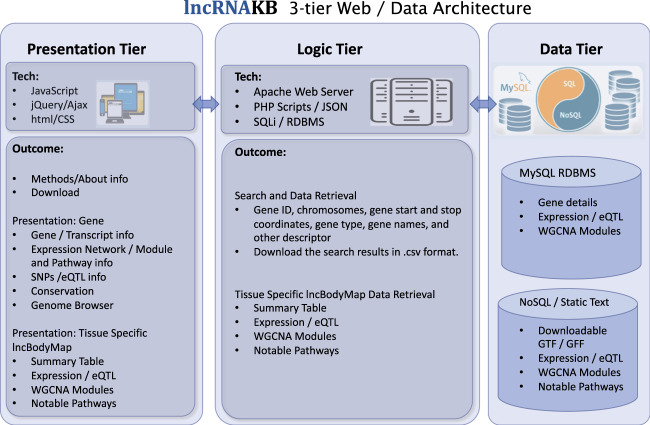
Schema of the web/database segment of the lncRNAKB.

The 3-tier server architecture model containing data, logic and presentation tiers has been implemented as shown in Fig. 3. The popular MySQL open source relational database management system (RDBMS) has been employed for the data tier, expanded with a NoSQL document storage. NoSQL document storage is a JSON-based (JavaScript Object Notation) data structure format and as such has a flexible dynamic structure with no schema constraints which makes it suitable for literature and document storage. The MySQL RDBMS is ideal for data indexing and a powerful query system for relational data. The logic tier is responsible for the communication between the user queries from the presentation tier and fetching the outcome from the data tier, as well as data integration from MySQL and NoSQL data sources. The presentation tier contains several modules based on AJAX (Asynchronous JavaScript and XML), jQuery (JavaScript Query system version 3.3.1 - https://jquery.com/), and the PHP server-side scripting language (version 7.1.18.), as well as the CSS (Cascading Style Sheets) code to describe how HTML elements are to be displayed on user side web interface. jQuery and AJAX have the advantage of asynchronous background calls to the logic tier, native JSON parsing, and dynamic rendering of the browser display, which makes the data retrieval system perform more efficiently. The Web server is hosted on a CentOS 7 operating system using an Apache (2.4.33) web server. The user interface is functional across major web-browsers such as Chrome, Safari, and Firefox on Linux, Mac, iOS, Android, and Windows OS platforms. All graphs are generated dynamically using Highcharts software and plotly^85^.

## Data Records

### Downloadable, searchable and viewable lncRNA annotation

Based on the PubMed search and literature review, six annotations were chosen to systematically integrate all the lncRNAs entries with the goal of providing one comprehensive annotation of lncRNAs (see Methods: Integration of lncRNA annotations).

CHESS was used as the reference annotation and contains protein-coding (*n = *20,352) and lncRNAs genes (*n = *18,897). CHESS already incorporated data from FANTOM, however, based on the cumulative stepwise intersection method we added additional 7,157 genes from FANTOM. LNCipedia on the other hand added 10,506 genes. NONCODE and MiTranscriptome added 20,700 and 15,164 genes respectively. While The last source, BIGTranscriptome, which annotates 13,525 records, contributed only 333 unique genes which indicates that there was extensive overlap with other annotations.

**Fig. 4:**
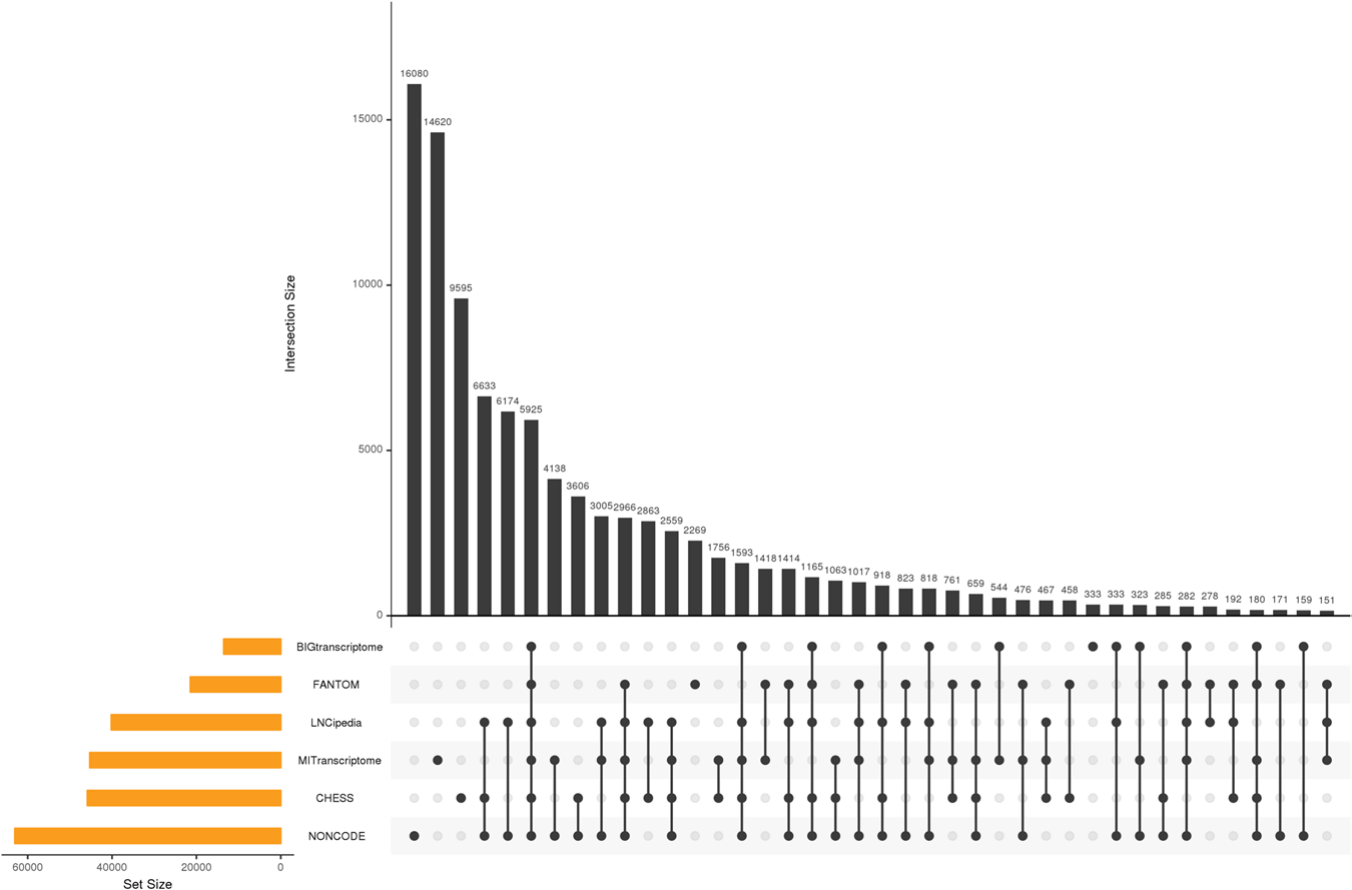
Upset plot showing the overlap of all six lncRNAs annotations at the gene level, after the cumulative stepwise intersection method across all. The orange bars indicate the total number of genes in each source before merging. The black bars indicate the total number of genes present within an annotation or shared between annotations indicated by black dots present below the x-axis of the plot. Genes uniquely contributed by a single annotation would be represented as a single dot that horizontally aligns with the respective annotation. Black dots connected by lines indicate the number of annotations that share the genes represented in the bar plot.

Figure 4 illustrates contribution of lncRNAs from each of the six annotations. It highlights that there was considerable overlap between different sub-sets of the annotations. All of LNCipedia genes overlapped with one or more of the other five annotations. NONCODE added the highest number of non-overlapping genes (*n = *16,080) followed by MiTranscriptome (*n = *14,620). BIGTranscriptome added only 333 unique gene entries due to sizeable overlap with others. CHESS was used as the reference annotation and contains protein-coding (*n = *20,352) and lncRNAs genes (*n = *18,897). However, from Fig. 4, we observed that the number of non-overlapping genes added from CHESS is 9,595, which indicates that we added non-coding transcripts from overlapping lncRNAs in other annotations to the protein-coding genes. 5,295 genes overlapped between all six sources. The number of transcript entries for the protein coding genes in lncRNAKB was much higher than that in CHESS (approximately 40,330 more transcript entries in lncRNAKB compared to CHESS). This suggests that a good proportion of the lncRNAs transcripts (~15%) overlap with or fall within the boundary of protein-coding genes. Figshare File F3^78^ shows the number of transcripts and the sources of annotations at gene level for non-coding genes between CHESS and lncRNAKB. It shows that we have effectively added numerous non-coding genes (*n = *77,199) and non-coding transcripts (*n = *224,286) from different lncRNAs annotations. In summary, the final merged annotation in lncRNAKB comprises of both protein-coding and lncRNA including 99,717 genes, 530,947 transcripts, and 3,513,069 exons.

The merged annotation of all the genes can be browsed via a searchable table or the GFF file can be downloaded from the website. Users can search lncRNAKB by common gene annotation IDs, chromosomes, gene start and stop coordinates, gene types, gene names, or any other descriptor. The results of the gene query are displayed in the gene page providing detailed information about the gene and displaying results from genomic analysis such as tissue-specific gene and transcript expression, tissue specificity score, eQTLs, network and pathway enrichment, trait associations, exon conservation scores and coding potential. A custom UCSC Genome Browser track showing all the transcripts and exons for that gene is also available. The annotations are hosted under the GTF/Annot component in OSF.

### Tissue-specific expression profiling of lncRNA

RNA-seq data from 31 tissues was accessed from GTEx. The data was processed using a custom RNA-seq analysis pipeline using the combined annotation file to establish the tissue-specificity of lncRNA (see Methods: Expression profiling). Figshare File F4^78^ shows the number of RNA-seq samples analyzed across 31 tissues (*n* = 9,425). Figshare File F5^78^ shows the summary statistics of alignment and quantification across all samples. Figshare File F1^78^ shows the distribution of uniquely aligned paired-end reads assigned to genes across all samples. Bars highlighted in red show the numbers of samples with <10^6^ reads assigned to genes (*n* = 351) that were excluded from further analysis. The expression matrices are hosted under the Expression component in OSF.

### Evaluating tissue-specificity of lncRNA

**Fig. 5:**
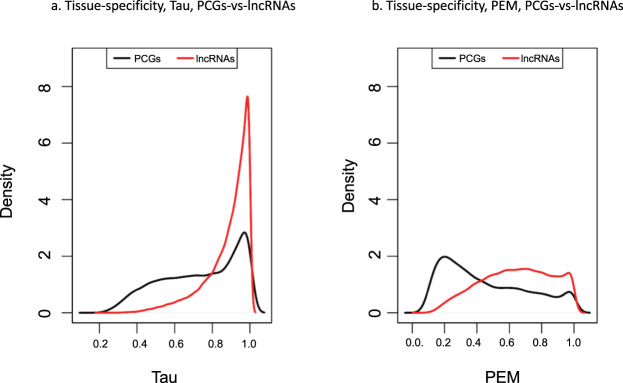
Distribution of tissue-specificity scores with data for RNA-seq from 31 solid human normal tissues from GTEx across protein-coding genes (PCGs) and lncRNAs in the lncRNAKB as a comparison. The tissue-specificity scores varies from 0 to 1, where 0 means broadly expressed, and 1 is specific. Graph created with density function from R, which computes kernel density estimates (**a)** Average Tau score across all tissues. (**b)** Maximum and normalized specificity value of PEM among all tissues.

Using the gene expression results described in the section above, the tissue- specificity score of all lncRNA was calculated. Two different metrics, Tau and Preferential Expression Measure (PEM), were calculated which illustrate the tissue-specificity of the lncRNA (see Methods: Tissue-specificity scores). Figure 5 shows the density distribution of tissue-specificity metrics Tau and PEM across protein-coding genes (PCGs) and lncRNA in the lncRNAKB annotation as a comparison. The tissue-specificity scores vary from 0 to 1, where 0 means broadly expressed, and 1 is specific. Figure 5a. displays average Tau score across all tissues and Fig. 5b. displays the maximum and normalized specificity value of PEM among all tissues.

### eQTL analysis of lncRNA

To add to our understanding of lncRNA gene expression information, we used the gene expression data (see Methods: Expression profiling) in combination with the whole genome sequencing (WGS) data available at GTEx to identify variants in the genome that can alter gene expression (see Methods: eQTL analysis). This analysis resulted in identification of a number of variants that significantly alter lncRNA gene expression in a tissue-specific manner. Table 2 summarizes the results of the *cis*-eQTL analysis. *Cis*-eQTL analysis was performed on 25 tissues that had >80 samples and accompanying WGS data. The WGS VCF file with 50,862,464 variants was processed and the resulting file had 5,835,187 SNPs that were used for the *cis*-eQTL analysis (see Methods: Genotype file processing). For each tissue, Table 3 summarizes the number of samples (stratified by sex), the number of SNPs available after pre-processing, the number of genes that met the TPM threshold criteria from the RNA-seq data (PCG and lncRNA), the total number of SNP-gene pairs that were tested within 1 Mb of the transcription start site (TSS) of each gene and the number of top *cis*-eQTL genes that met FDR ≤0.05 threshold. (see Methods: eQTL analysis). The eQTL results are hosted under the eQTL component in OSF.

**Table 2:** Summary results of the *cis*-eQTL results available from lncRNAKB. Tissues with <80 samples are shown here but, were excluded from the analysis.

Tissue	Number_of_RNA_seq_samples_with_WGS	Number_of_Males	Number_of_Females	Number_of_SNPs_with_MAF_greater_than_0.05	Total_number_of_genes_passed_filter	Total_number_of_PCGs	Total_number_of_lncRNAs	Total_SNP_gene_pairs_eQTLs	Total_SNP_gene_pairs_with_permutation_pvalue_less_than_0.05
Adipose_Tissue	363	220	143	5,952,169	27,029	15,175	11,854	54,871,184	5,766
Adrenal_Gland	146	82	64	5,886,806	25,943	14,973	10,970	51,879,876	4,077
Bladder	9	4	5	5,462,615	28,695	15,597	13,098	*	*
Blood	356	226	130	5,953,536	18,412	11,788	6,624	37,414,178	2,877
Blood_Vessel	378	241	137	5,963,536	25,614	14,770	10,844	51,947,442	5,854
Bone_Marrow	*	*	*	*	22,571	12,612	9,959	*	*
Brain	170	116	54	5,857,467	31,339	16,148	15,191	62,844,553	3,488
Breast	184	102	82	5,901,708	28,839	15,680	13,159	58,130,064	4,267
Cervix_Uteri	8	0	8	5,522,234	28,706	15,649	13,057	*	*
Colon	250	148	102	5,907,992	28,297	15,781	12,516	57,063,773	4,767
Esophagus	353	221	132	5,941,386	26,803	15,439	11,364	54,314,052	4,815
Fallopian_Tube	7	0	7	*	18,492	16,552	1,940	*	*
Heart	251	163	88	5,913,705	24,959	14,788	10,171	50,153,256	4,375
Kidney	29	23	6	5,742,588	28,917	15,726	13,191	*	*
Liver	118	77	41	5,871,833	23,846	14,204	9,642	47,689,780	2,759
Lung	274	182	92	5,926,605	29,045	15,744	13,301	58,884,074	5,461
Muscle	359	220	139	5,962,131	22,042	13,558	8,484	44,548,539	4,454
Nerve	268	174	94	5,941,274	29,326	15,472	13,854	59,363,204	7,416
Ovary	99	0	99	5,873,449	27,292	14,845	12,447	54,588,663	3,466
Pancreas	167	98	69	5,905,087	23,569	14,210	9,359	47,408,959	*
Pituitary	108	76	32	5,814,865	30,586	15,848	14,738	60,707,019	3,949
Prostate	101	0	101	5,810,666	30,373	15,931	14,442	60,377,553	*
Salivary_Gland	63	43	20	5,771,591	28,409	15,679	12,730	*	*
Skin	442	278	164	5,966,760	27,316	15,442	11,874	55,698,051	6,210
Small_Intestine	90	54	36	5,777,092	30,046	15,950	14,096	59,426,622	2,987
Spleen	108	62	46	5,874,443	28,284	14,969	13,315	56,914,604	4,743
Stomach	182	104	78	5,890,077	26,974	15,530	11,444	54,242,450	3,804
Testis	171	0	171	5,875,543	47,909	17,777	30,132	98,376,057	8,951
Thyroid	286	183	103	5,941,584	29,715	15,604	14,111	60,217,108	7,611
Uterus	82	0	82	5,795,583	28,175	15,166	13,009	55,748,102	3,037
Vagina	87	0	87	5,837,620	28,423	15,629	12,794	56,861,978	2,865

**Table 3:** Summary of classification of lncRNA transcripts with respect to their localization, overlap and orientation relative to transcription of proximal protein-coding RNA transcripts.

	^1a^Overlapping	^1^GENIC	^1c^Nested	Total
		^1b^Containing		
Antisense Exonic	9,326	1,816	3,552	14,694
Antisense Intronic	1,302	1,284	8,330	10,916
Sense Exonic	29,942	42,160	29,087	101,189
Sense Intronic	327	994	13,274	14,595
Total	40,897	46,254	54,243	141,394
		^**2**^**INTERGENIC**		
	^**2a**^**Convergent**	^**2b**^**Divergent**	^**2c**^**Same_Strand**	**Total**
Upstream	—	14,930	13,408	26,470
Downstream	11,540	—	10,662	24,070
Total	11,540	14,930	24,070	50,540

The legend below explains the categories in detail:

^1^GENIC: when the lncRNA gene overlaps an RNA gene from the reference annotation file

^2^INTERGENIC (lincRNA): otherwise.

GENIC type:

Then exonic or intronic locations:

^1a^Overlapping subtype: the lncRNA partially overlaps the RNA partner transcript.

^1b^Containing subtype: the lncRNA contains the RNA partner transcript.

^1c^Nested subtype: the lncRNA is contained in the RNA partner transcript.

INTERGENIC type:

^2a^Divergent subtype: the lncRNA is transcribed in head to head orientation with RNA partner transcript: upstream or downstream.

^2b^Convergent subtype: the lncRNA is oriented in tail to tail with orientation with RNA partner transcript: upstream or downstream.

^2c^Same_strand subtype: the lncRNA is transcribed in the same orientation with RNA partner transcript: upstream or downstream.

### Functional characterization of lncRNA using a network-based approach

To further our understanding of potential lncRNA function, we also undertook WGCNA, a network-based approach that relies on calculating correlation of expression between genes and identifying clusters/modules of genes (both protein-coding and lncRNA) with similar expression patterns (see Methods: Functional characterization of lncRNA using a network-based approach). Since correlated genes are predicted to play similar functions in the cells, the pathway enrichment analysis of the correlated clusters/modules can help characterize the potential functions of lncRNA in the correlated module. Figshare File F6^78^ summarizes the results of the WGCNA analysis across the 28 tissues using the GTEx RNA-seq data. WGCNA analysis was not performed on three tissues (Bladder, Cervix_Uteri and Fallopian_Tube) due to insufficient sample size. After filtering genes with low expression (see Methods: Functional characterization of lncRNA using a network-based approach), the average number of protein-coding genes was 14,699 and lncRNA was 3,389, per tissue. We identified total of 1,208 lncRNA-mRNA co-expression modules across all tissues (on average approximately 43 modules per tissue). On average, across all tissues, each module had approximately 487 genes including 92 lncRNA, indicating favourable co-expression of lncRNA with PCGs. Figshare File F6^78^ also summarizes the results of the over-representation analysis (ORA) based on the hypergeometric test using the Gene Ontology (GO) terms across all the modules identified. It displays the number of GO terms tested, number of terms with p-value ≤0.05 and FDR q-value ≤0.05 in all modules by tissue. On average, across all modules, each tissue had approximately 2,592 pathways with q-value ≤0.05, indicating significant enrichment of biological processes within each of these modules. The WGCNA results are hosted under the WGCNA component in OSF.

### lncRNA-trait associations

To systemically map human lncRNA regulated by the eQTLs that colocalize with GWAS loci of diseases or traits we used the *cis*-eQTL and UK Biobank GWAS data (323 traits >5,000 cases). Using SMR analysis we determined if our identified *cis*-eQTLs of lncRNA were functionally colocalized with the GWAS signals. Due to complicated linkage disequilibrium between variants in the human genome, we applied the method of HEIDI implemented in SMR. Figshare File F2^78^ summarizes the results of the SMR analysis in 25 tissues across all traits. For each tissue, it shows the number of genes with pSMR ≤0.05 (genes prioritized by SMR) across all traits. The SMR results are hosted under the Trait Association component in OSF.

### Evaluation of coding potential of lncRNA

To characterize the lncRNA annotated in lncRNAKB, FEELnc algorithm was used to classify them based on their position, and their coding potential was evaluated. After applying the FEELnc filters (removing transcripts <200 bp long and >75% overlap with protein-coding transcripts, (see Methods: Evaluation of coding potential of lncRNA), the lncRNAKB GFF annotation file resulted in 96,539 genes, 311,241 transcripts and 1,200,236 exons that were considered to be “candidate lncRNA.” The coding potential score (CPS) cut-off determined by the Random Forest (RF) classification on the training data was 0.434 (separating protein-coding (mRNA) versus lncRNA transcripts) with an Area Under the Curve (AUC) performance of 0.972 which maximizes the mRNA classification sensitivity and specificity (see Methods: Evaluation of coding potential of lncRNA). Based on this cut-off, 83,190 genes, 219,324 transcripts were classified as lncRNA and 31,402 genes, 91,845 transcripts as protein-coding. The classification module categorized 141,394 lncRNA transcripts as GENIC (when the lncRNA transcript overlaps an mRNA/protein-coding transcript from the reference annotation file) and 50,540 as INTERGENIC (lincRNA). Several lncRNA transcripts did not have an interacting mRNA partner thus, remained positionally unclassified. Table 3 summarizes the results of the classifier module with a breakdown of interactions between the two types of lncRNA and their partner mRNA/protein-coding transcripts. The lincRNA are, on average 23 kb away from their mRNA partner.

### Evaluation and comparison of lncRNA and mRNA conservation scores

**Fig. 6:**
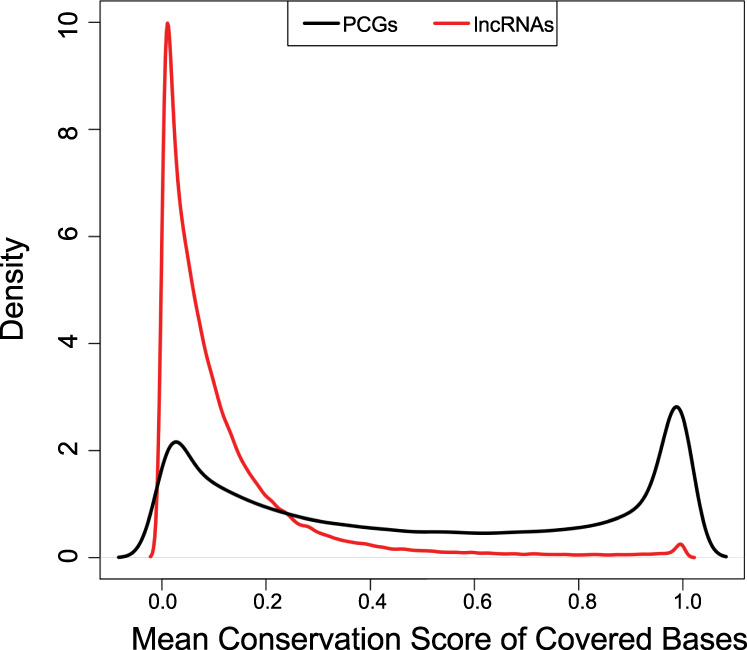
Distribution of mean PhastCons exon sequence conservation scores across lncRNA and protein-coding genes in the lncRNAKB. Graph created with density function from R, which computes kernel density estimates.

In addition to evaluating the coding potential, the conservation of exonic sequences of the lncRNA and mRNA was determined (see Methods: Conservation analysis) and compared. Figure 6 shows the density distributions of exon sequence conservation scores comparing protein-coding genes (PCGs) and lncRNA in the lncRNAKB annotation. Overall, it shows that exons of the PCGs have higher mean sequence conservation scores compared to exons of the lncRNA.

## Data Download

The datasets generated and/or analysed during the current study are available on lncRNAKB website (http://lncrnakb.org) as well as through Open Science Framework (10.17605/OSF.IO/RU4D2)^44^.

All supplementary data are available from Figshare (10.6084/m9.figshare.12563864.v3)^78^

## Technical Validation

**Fig. 7:**
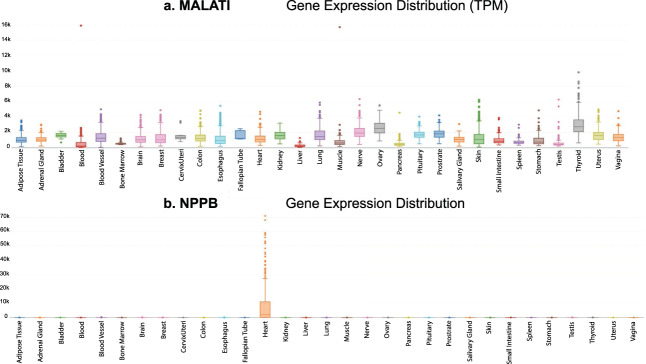
Gene expression box plot distributions of gene (**a)**. *MALAT1* (Metastasis Associated Lung Adenocarcinoma Transcript 1) and (**b)**. *NPPB* (natriuretic peptide B). The x-axis represents the 31 solid human normal tissues from GTEx and y-axis is the TPM expression.

Figure 7a, b. visualizes two gene expression distribution box plots of *MALAT1* (Metastasis Associated Lung Adenocarcinoma Transcript 1) and *NPPB* (natriuretic peptide B) respectively. *MALAT1* is a widely studied lncRNA expressed in all tissues but, specific to the following as shown by the PEM scores distribution (colon, blood vessel, vagina, bladder, fallopian tube, kidney, cervix/uteri, lung, pituitary, uterus, prostate, nerve, ovary and thyroid), ranging from 0.01–0.35 on lncRNAKB (see Methods: Tissue-specificity scores). According to the lncRNA and disease database^86^ (http://www.rnanut.net/lncrnadisease/) it is involved in multiple cancers such as bladder, breast, cervical, colorectal, kidney, liver and lung. In addition, the trait association results on lncRNAKB indicate lung and bowel cancer in which *MALAT1* is prioritized at pSMR ≤0.05. *NPPB* is a PCG with a PEM score of 1.49 in the heart tissue (specific to only the heart). It functions as a cardiac hormone and plays a key role in cardiac homeostasis^87^. A high concentration of this protein in the bloodstream is indicative of heart failure. Even though *NPPB* is categorized as a PCG, it has five transcript isoforms that were classified as lncRNA. The trait association results of *NPPB* indicate many heart related conditions in which it is prioritized at pSMR ≤0.05.

**Fig. 8:**
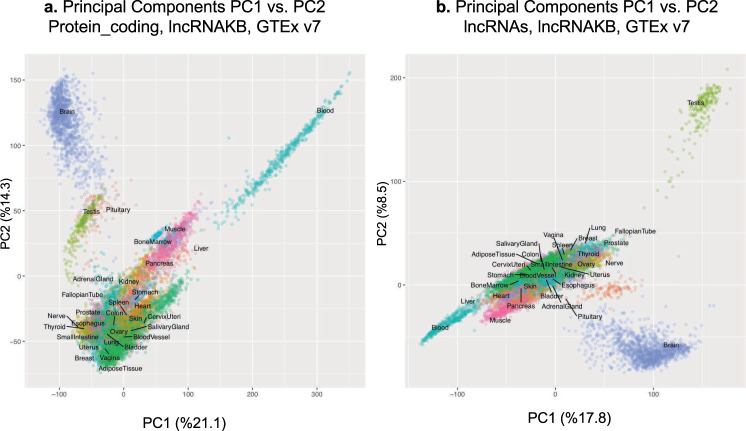
Principal Component Analysis (PCA) of GTEx samples using (**a**). protein-coding and (**b**). lncRNA (*log*_2_(*TPM*) transformed gene expression. Expression of lncRNA alone also recapitulates tissue types.

To validate the annotation and the expression profiling analysis, we performed an unsupervised principle component analysis (PCA) of the gene expression data separately for lncRNA and mRNA (see Methods: Expression profiling). For this analysis, the log transformed TPM lncRNA and mRNA expression data across all tissues was used. Each tissue showed a characteristic transcriptional signature, as revealed by PCA of lncRNA and mRNA expression. The separation was evident between blood and other tissues whilst brain and testis were the most distinct (protein-coding and lncRNA, Fig. 8a,b., respectively). This finding was an additional confirmation that mRNA are tissue-specific whereas lncRNA expression can distinguish tissues as well.

**Fig. 9:**
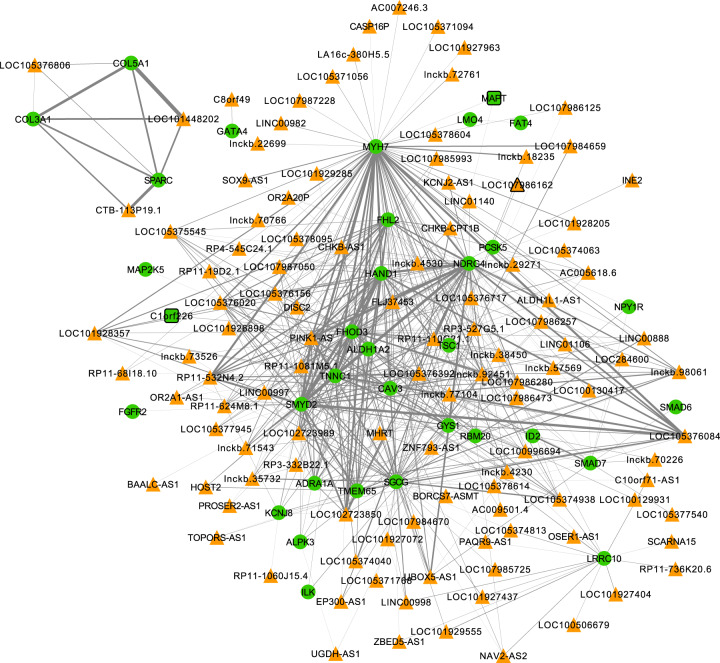
Cytoscape network for lncRNA-mRNA co-expression Module 2 (M2) in the heart identified using WGCNA. The network was filtered for heart development genes (*n* = 148) and correlations >0.20. Orange triangles and green circles/nodes represent lncRNAs and PCGs respectively. The density of gray lines/edges represents the strength of the connection between genes.

To validate the functional characterization of lncRNA, there were 61 modules identified in the heart using gene expression data across 16,882 protein-coding genes and 2,762 lncRNA (network and pathway enrichment data available in the knowledgebase). There were several significant GO terms enriched (q-value <  = 0.05) with many of these involved in heart related biological processes. Figure 9 highlights the network figure created using Cytoscape for module M2 identified in the heart tissue. This module is involved in heart-specific processes such as heart growth, development and contraction. The network has 148 genes (34 protein-coding and 106 lncRNA) after filtering the adjacency matrix with correlations <0.20 and “heart development” specific pathways/genes. The orange triangles and green circles/nodes represent lncRNA and mRNA respectively. The thickness of the edges highlights the correlation between nodes. The relatively strong connections of several lncRNA to PCGs in this network suggests these could be potentially involved in the same heart development specific biological processes.

## Usage Notes

Below is a brief tutorial explaining how to navigate through the data and several components on the lncRNAKB website. We have created a How To page that contains detailed video tutorials on sections of lncRNAKB and how to navigate through the available data. In addition, we plan to update the data once in every six months or when there are significant changes in the integrated lncRNA annotations.

### Browse gene

On the Browse Gene page, users can search for any gene of interest using multiple criteria. The information below is provided for each searched gene.

#### Gene info

On the gene page, users will get annotation information on the gene (including the original source of the annotation and the gene type i.e. protein coding, lncRNA, antisense or miscellaneous RNA). The annotation information for that gene can be downloaded by clicking on the image icons. A downloadable text and CSV file with transcript and exon records of the gene from the GFF annotation is provided as well as a snapshot image from the UCSC genome browser with a custom track created using the lncRNAKB GFF annotation.

#### Tissue expression

To visualize the gene expression levels, users can view or download dynamic boxplots or expression matrices of TPM across 31 tissues.

#### Tissue specificity

The distribution of PEM scores in a given tissue in relation to its average expression across all other genes and other tissues can be viewed or downloaded using dynamic bar charts or PEM score matrices across 31 tissues.

#### Network and pathway

A dynamic table containing the top three over-represented Gene Ontology pathways in which the gene is a member of a co-expression module is displayed or can be downloaded. Users can click on the tissue of interest to navigate to the specific tissue page, click on the pathway of interest to go to the pathway description page in MSigDB, download the adjacency matrix of each module or download the full pathway enrichment results by clicking on the CSV icon next to the tissue.

#### eQTL

A dynamic barplot showing the number of SNPs that alter the expression of the gene at pvalue <0.05 for the indicated tissues are summarized, with the number of SNPs altering the expression printed on the respective bars on the barplot. A List of 1,000 SNPs that alter the expression of the gene for the indicated tissues are shown in a dynamic table and the complete results (pvalue <0.05) can be downloaded. By clicking on the tissue, users can navigate to the specific tissue page to download the full eQTL results.

#### Transcript

A dynamic table displaying all the transcripts in the gene. Shown in the table below is the positional classification and the coding potential of all the transcripts for the gene. To visualize the gene expression levels by transcript, users can click on the transcript ids to view or download dynamic boxplots or expression matrices of TPM across 31 tissues. Additionally, the conservation scores for all the exons (overlapping exons merged) in a gene are shown in a dynamic table.

#### Trait association

A dynamic table displaying the list of traits in which the gene was prioritized for the indicated trait in specific tissues is shown. By clicking on phenotype IDs, information about the phenotypes are provided through the UK Biobank. By clicking on phenotype names, a dynamic bar chart is generated showing the number of genes with pSMR ≤0.05 across all tissues. By clicking on the tissue, users can navigate to the specific tissue page to download the trait association results with pSMR ≤0.05.

#### Genome browser

A fully functional UCSC genome browser is displayed with a custom track of the gene annotation illustrating the transcripts and exons from the lncRNAKB GFF annotation.

#### Gene expression

On the Gene Expression page, users can download genome-wide expression matrices (raw counts and TPM) at the gene and transcript level, quantified using the lncRNAKB GFF annotation as well as quality control data for alignment and quantification across all samples in text format by tissue.

### eQTL

On the eQTL page, users can view and download the *cis*-eQTL results via Manhattan plots and genome-wide *cis*-eQTL results (all SNP-gene pairs) in text format by tissue. FDR corrected pvalues are included in each file.

### Trait association

On the trait association page, users can view all the traits (*n* = 323) analyzed using SMR as a dynamic table. By clicking on phenotype IDs, information about the phenotypes are provided through the UK Biobank. By clicking on phenotype names, a dynamic bar chart is generated showing the number of genes with pSMR ≤0.05 across all tissues. Users can click on a tissue on the bar chart and navigate to the SMR results page for that trait, displayed as a dynamic table including genes prioritized with pSMR ≤0.05. By clicking on a gene id, information on that gene (described in the Browse Gene section above) is shown. By clicking on dbSNP rsIDs, information about SNPs are provided through dbSNP.

### lncBodyMap

On the body map page, users can click on the tissue of interest to view and download tissue-specific gene expression, eQTL, trait association, network and pathway enrichment results.

#### Network and pathway enrichments

For each tissue, a graphical summary of the WGCNA results are displayed. It shows interesting pathways in which different genes part of distinct co-expression modules are overrepresented. A dynamic table showing the top 1,000 significant pathways (qvalue ≤ 0.05) are displayed and the full list of significant pathways can be downloaded. All pathways enrichment results across all modules can be downloaded as well as the adjacency matrices by module. In addition, 25 notable pathways were selected for each tissue and network files highlighting the lncRNA-mRNA correlations were generated. Users can visualize and download the corresponding dynamic network figures and review the connections between lncRNA and mRNA involved in selective biological processes of interest.

### Download

Users can download the comprehensive GFF file across all genes or lncRNA only.

### Methods

Users can refer to online methods for further details of the analysis.

## Data Availability

All code used to perform the analysis for data displayed and deposited on lncRNAKB is available through https://github.com/seifudd/lncRNAKB
